# Controlled doping by self-assembled dendrimer-like macromolecules

**DOI:** 10.1038/srep41299

**Published:** 2017-02-01

**Authors:** Haigang Wu, Bin Guan, Yingri Sun, Yiping Zhu, Yaping Dan

**Affiliations:** 1 School of Biomedical Engineering, Shanghai Jiao Tong University, Shanghai, 200240, China; 2 University of Michigan-Shanghai Jiao Tong University Joint Institute, Shanghai Jiao Tong University, Shanghai, 200240, China; 3 Key Laboratory of Polar Materials and Devices, Ministry of Education, and Department of Electronic Engineering, East China Normal University, Shanghai 200241, China

## Abstract

Doping *via* self-assembled macromolecules might offer a solution for developing single atom electronics by precisely placing individual dopants at arbitrary location to meet the requirement for circuit design. Here we synthesize dendrimer-like polyglycerol macromolecules with each carrying one phosphorus atom in the core. The macromolecules are immobilized by the coupling reagent onto silicon surfaces that are pre-modified with a monolayer of undecylenic acid. Nuclear magnetic resonance (NMR) and X-ray photoelectron spectroscopy (XPS) are employed to characterize the synthesized macromolecules and the modified silicon surfaces, respectively. After rapid thermal annealing, the phosphorus atoms carried by the macromolecules diffuse into the silicon substrate, forming dopants at a concentration of 10^17^ cm^−3^. Low-temperature Hall effect measurements reveal that the ionization process is rather complicated. Unlike the widely reported simple ionization of phosphorus dopants, nitrogen and carbon are also involved in the electronic activities in the monolayer doped silicon.

Precisely placing individual dopants at arbitrary location is critical for single atom electronics^1^ and sub-nanometer integrated circuits^1^,^2^. Single-ion implantation technique^2^,^3^ and hydrogen lithography based on scanning tunneling microscopy (STM)^4^,^5^ have been successfully developed to control single dopants. However, these techniques are time-consuming serial processes in which dopants are placed one by one. A parallel process for control of individual dopants at large scale is required for industrial applications^6^. Self-assembled monolayer (SAM) doping might offer a promising solution if the dopant-carrier molecules can be individually controlled^7^. Up to date, the SAM doping technique mostly uses small molecules for the purpose of achieving ultra-shallow junctions^9^,^10^. It is extremely challenging to control the location of individual carrier molecules unless the molecule size is large enough. Recently, Popere *et al*. reported controlling the locations of dopants by using self-assembled block copolymers with dopants encapsulation^11^. The self-assembled nature of block copolymers allows for placing dopants in a periodic array. Although the periodic distribution of dopants is useful in, for instance, minimizing the sub-threshold voltage fluctuation in deep nanoscale field-effect transistors^2^, it cannot meet the requirements for circuit design that requires the individual dopants in single atom electronics to be placed at arbitrary locations.

To exclude any potential electrical interference, the best candidate for the carrier molecule is the one that is large (at least a few nanometer in diameter to be comparable to the resolution of the most advanced lithography) and mostly importantly, only has C, O and H in addition to the dopant element such as P or B since C, O and H do not electrically dope silicon^12^,^13^. Here, we demonstrate a novel monolayer doping approach by using the super-branched poly-glycerol molecules that are synthesized to carry only one phosphorus atom in the core (Fig. 1)^14^,^15^. The synthesized macromolecules are then grafted onto the silicon substrate *via* the reaction with carboxyl-functionalized silicon surfaces. The electrical and secondary ion mass spectrometry (SIMS) measurements indicate that phosphorus dopants have successfully diffused into and electrically doped the silicon substrate after rapid thermal annealing. Low-temperature Hall effect measurements are employed to further investigate the ionization of the dopants in the samples.

## Result and Discussion

### Synthesis of Polyglycerol

**Figure 2:**
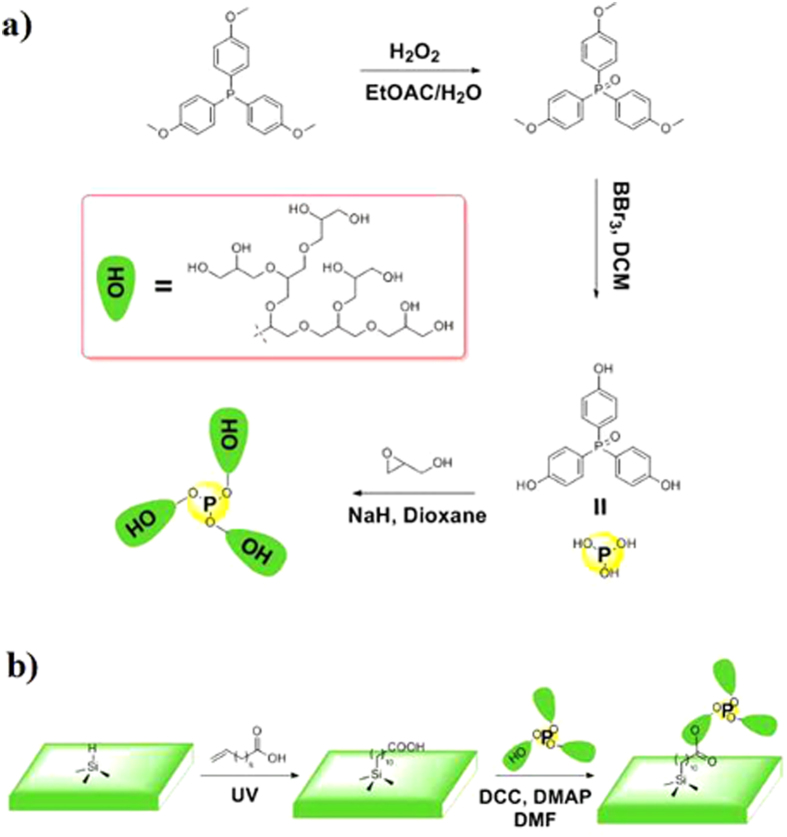
(**a**) Synthesis of the phosphorus carrier core and polyglycerol. (**b**) Silicon surface modification with undecylenic acid followed by immobilization of polyglycerol onto silicon surface by coupling reagents.

To synthesize macromolecules with each carrying one phosphorus atom, we choose tris(4-methoxyphenyl)phosphine (TMP) as the core to initiate the polymerization with glycerol. Note that the three-valence phosphine in TMP can be easily oxidized during the demethylation process. To avoid creating other byproducts, TMP was first oxidized as shown in the synthesis route in Fig. 2a. In the polymerization process, the molecular molar ratio of glycerol to tris(4-methoxyphenyl)phosphine oxide was set to 450:1 to reach the size of up to several nanometers in diameter for polyglycerol as shown in the literature reports^16^.

After the polymerization, the product was purified in the dialysis bag (Cut-off molecular weight > 1000 g·mol^−1^) to remove possible light-weight molecules. Gel Permeation Chromatography (GPC) was applied to characterize the molecular weight of the filtered polyglycerol (Fig. S1 in Supporting Information (SI)). The GPC results indicate that the weight- (*M*_w_) and number-average (*M*_n_) molecular weight are 10296.4 g·mol^−1^ and 5452.2 g·mol^−1^, respectively. The resultant *M*_w_/*M*_n_ ratio is ~1.89, implying that the molecular weight profile has low polydispersity indices. Fourier Transform Mass Spectrometry-Electron Spray Ionization (FTMS-ESI) was employed to scan the molecular weight region from 1000 g·mol^−1^ to 5000 g·mol^−1^. The results (SI Figs S2 and S3) show that the produced polymers with molecular weight lower than 1000 g·mol^−1^ are not observed, in agreement with the dialysis process.

**Figure 3:**
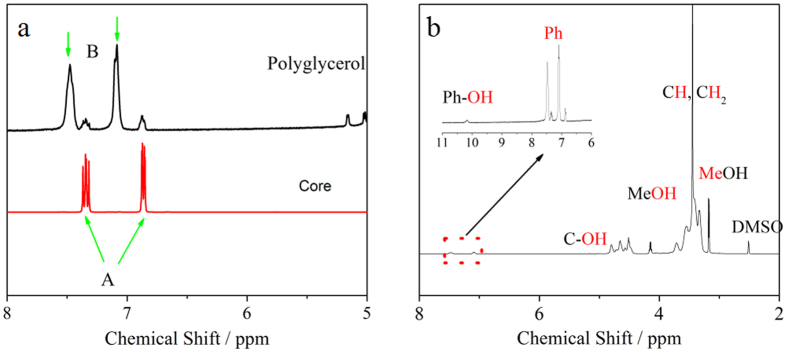
(**a**) Comparison of ^1^H NMR spectrum of polyglycerol and core between 5 ppm and 8 ppm. (**b**) ^1^H NMR spectrum of polyglycerol. The signature of phenyl ring is amplified in the inset.

A series of nuclear magnetic resonance spectroscopy (NMR) techniques, including proton, carbon-13 and phosphorus-31 NMR, were utilized to verify the structure of the polyglycerol. ^1^H NMR was first applied to check whether the macromolecules carry the tris(4-hydroxyphenyl)-phosphine (TMP) oxide which is the core of the macromolecule. Four peaks are observed in the spectrum from 6 ppm to 8 ppm (7.48 ppm, 7.34 ppm, 7.09 ppm and 6.88 ppm) as showed in Fig. 3a (black line), which are identified as the signature of aromatic rings. The macromolecule is formed by polymerizing the glycerol moieties at the three phenyl terminals of TMP oxide. Due to the complexity and randomness of the reaction, some of the phenyl terminals may be left unreacted. The two small peaks at 7.34 ppm and 6.88 ppm are from these unreacted phenyl groups, since two peaks at exactly the same locations are observed for pure TMP oxide (red curve). Note that small molecules such as unreacted pure TMP oxide are not possibly left in the final substance because small molecules would have been removed by dialysis bag filtering as previously described. The other two peaks at 7.48 ppm and 7.09 ppm belong to the reacted phenyl groups, which are left-shifted to low field likely due to the decrease in electron density of phenyl groups after substitution of glycerol moieties at the phenyl terminals. Similar left-shift in the ^31^P NMR spectrum was also observed for TMP oxide after reacted with glycidol (SI Figs S4–S5). The existence of phenyl core in the macromolecules was further verified by ^13^C Distortionless Enhancement by Polarization Transfer (DEPT) 135° NMR (SI Fig. S6). Further analysis of the two sets of peaks for reacted and unreacted phenyl groups (black line in Fig. 3a) indicates that the two peaks for the reacted are also larger in peak area in addition to the aforementioned left-shift phenomenon. Quantitatively, the peak area ratio of unreacted to reacted phenyl groups is 0.11 (Fig. 3a), meaning that only 9.9% of phenyl terminals has not reacted with glycidol.

Chemical signature of the glycerol after polymerization shows up in the higher field region of ^1^H NMR spectrum in Fig. 3b. The peaks are labeled based on the analysis of the macromolecule’s structure^17^. The sharp spike with several shoulders between 3.2 ppm and 3.8 ppm belong to the carbon backbone of the polyether which contains -CH- and -CH_2_-. The three bumps between 4.4 ppm and 5.0 ppm are related to the hydroxyl in the structures of *L*_*13*_, *L*_*14*_ and *T* where the *L*_*13*_, *L*_*14*_ and *T* represent the branching structures to be discussed in next paragraph. Peaks at 2.5 ppm and 3.3 ppm are from the deuterium reagent DMSO-*d*_*6*_ and peaks at 3.2 ppm and 4.2 ppm from methanol solvent. A tiny kink at 10.1 ppm is believed to be the signature of the hydroxyl of un-reacted phenyl group (inset in Fig. 3b), while the four peaks at right side of the kink have been discussed in the previous paragraph. The existence of diversity of hydroxyl groups shows that some hydroxyl groups are not completely super-branched except at branch terminal.

**Figure 4:**
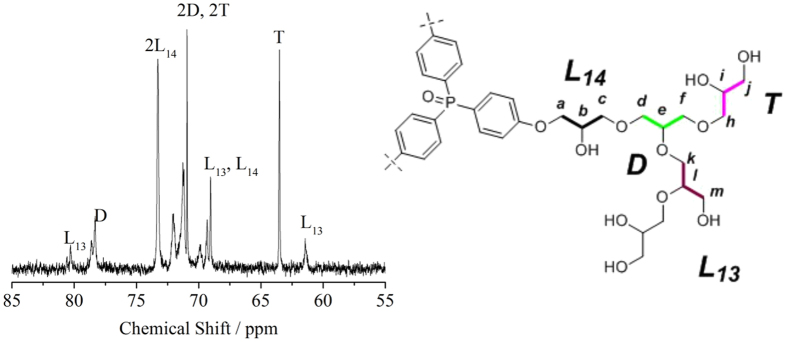
Inverse gated (IG) ^13^C NMR of macromolecule (left) and molecular structure of polyglycerol (right).

Inverse gated (IG) ^13^C NMR (Fig. 4) along with the ^13^C DEPT 135° NMR (SI Fig. S6) was applied to further check the branching structure of the synthesized polymer. Signals from dendritic (*D*), terminal (*T*), and linear (*L*) structural features are identified in relatively low field from 60 ppm to 80 ppm according to previous literature reports^17^. The *D* peak is from the branched hydroxyl groups of monomer glycerol (Fig. 4 right *d,e,f*). The *T* spike is related to the terminals of polyglycerol containing two free hydroxyl groups (Fig. 4 right *h,i,j*). The *L*_13_ (Fig. 4 right *k,l,m*) and *L*_14_ (Fig. 4 right *a,b,c*) signals are associated with the locations of free hydroxyl groups in the carbon chain^18^. The degree of branching (*DB*) for the synthesized polyglycerol macromolecules is determined by the integral area of the *D, T, L*_13_ and *L*_14_ peaks in Table 1 (calculation based on Frey’s equation: DB = *2D*/(*L*_*13*_ + *L*_*14*_ + *2D*)). The resultant *DB* of 0.66 indicates that the polymer is super branched (average dendrimer generation based on *M*_n_ is about 5.15)^19^. In short, these results indicate that polyglycerol is well super-branched and successfully synthesized as a phosphorus carrier.

### Silicon Surface Modification

**Figure 5:**
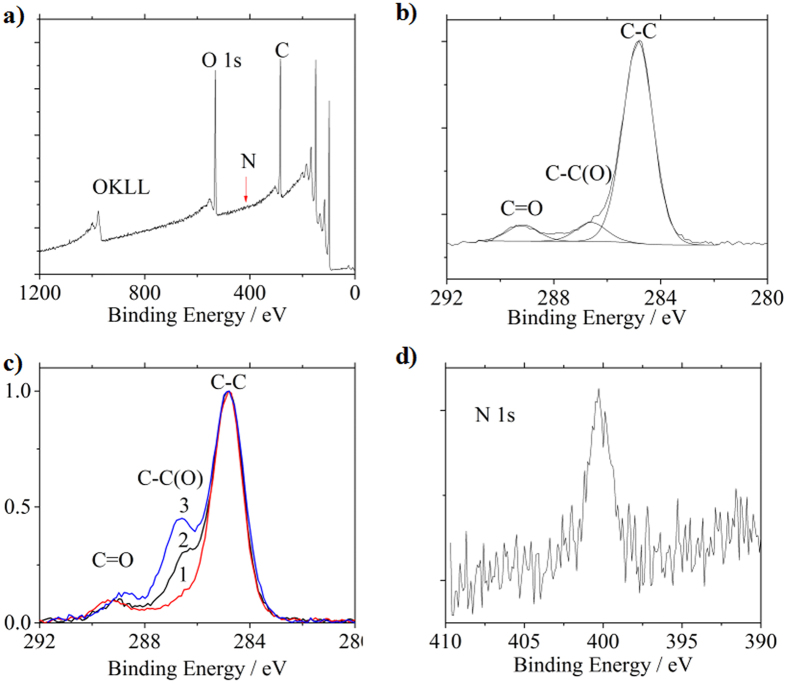
XPS spectra of functionalized silicon samples. (**a**) Survey spectra of the “11 Days” sample. (**b**) C 1s region for the control sample (without DCC). (**c**) Normalized C 1s scans for the control (“1”), 2 Days (“2”) and 11 Days (“3”) samples. (**d**) N 1s narrow scan for the “11 Days” sample.

To graft the synthesized polyglycerol onto silicon surfaces, the surfaces of silicon (nominal resistivity > 10,000 Ω·cm) were firstly modified with a monolayer of undecylenic acid *via* UV-induced hydrosilylation under inert atmosphere for 16 hours^20^. The carboxyl-functionalized silicon surfaces were characterized by high-resolution X-ray photoelectron spectroscopy (XPS). By analyzing the Si(-O_x_) and Si 2p peak, we conclude that a high-quality monolayer of undecylenic acid was successfully immobilized on the silicon surface since the silicon surface is well protected from being oxidized (only a fractional 0.2 monolayer of native SiO_x_ was observed. See details in SI Fig. S7). After the modification of undecylenic acid, the synthesized polyglycerol was then anchored at the presence of the coupling reagent dicyclohexylcarbodiimide (DCC) to the surfaces *via* the reaction with the carboxyl group (Fig. 2b). To investigate the polyglycerol binding efficiency, the reaction time for some samples was extended from 2 days (the “2 days” sample) to 11 days (the “11 days” sample) at room temperature. In the sample preparation process, one sample without the presence of DCC was also prepared at the same condition for reference (control sample). XPS was carried out again to characterize the silicon surfaces following the polyglycerol grafting (Fig. 5a).

On the polyglycerol grafted silicon surface, the majority carbon atoms are bonded as C-C(O) (including both C-C=O and C-C-O in which the C-C binding energy is the same) while on the undecylenic acid modified surface, only a small portion of carbon atoms exists in this form^21^. This structural contrast allows the C-C(O) peak to act as a marker to identify the polyglycerol binding on the silicon surface. Let us first take a look at the XPS spectrum of the control sample (Fig. 5b). The spectrum is decomposed by fitting into the C-C peak at 284.8 eV, the C=O peak at 286.4 eV and the C-C(O) peak at 288.9 eV. The characteristic C-C(O) peak is ~1.2 times of the C=O peak in terms of integral area, which is close to the stoichiometric ratio (1:1) of C-C(O) to C=O in undecylenic acid molecules. A similar ratio (0.98) has been observed for silicon surfaces modified only with undecylenic acid monolayers (SI Fig. S8). This observation indicates that nearly no polyglycerol molecules are bonded onto the control sample surfaces (without DCC). In contrast, as the polyglycerol reaction time (with DCC) is extended from 2 days (curve “2” in Fig. 5c) to 11 days (curve “3” in Fig. 5c), the relative strength of C-C(O) peak at 286.4 eV monotonically increases respective to the control sample (curve “1”) as shown in Fig. 5c. It is evident that polyglycerol molecules have successfully been bonded to the undecylenic acid on the silicon surfaces. Quantitatively, the integral area ratio of C-C(O) to C=O peak is 4.3 for the “11 days” sample (curve 3), indicating that the contribution of the PG macromolecules to the ratio is 3.1 (after deducting 1.2 which is the contribution of undecylenic acid). Since the number average molecular weight (M_n_) of PG molecule is 5452.2 g/mol, the number of C-C(O) bonds per PG molecule is ~58 on basis of the molecular structure. As a result, the ratio of PG to undecylenic acid molecules is approximately 1:22.

Since phosphorus atom is located in the center of the polyglycerol macromolecule and the relative quantity of phosphorus is low compared to carbon and oxygen, it is not surprising that nearly no phosphorus signal is observed in P 2p region (from 117 eV to 141 eV, SI Fig. S9) and P 2 s region (from 177 eV to 195 eV, SI Fig. S10) in the spectrum. Unexpectedly, we do observe a weak N 1s peak around 401 eV (400.3 eV in Fig. 2a and d, attributed to N from amide^22^) which is most likely originated from the coupling reagent DCC as explained in the following. In the process of ester formation with the DCC reagent (SI Fig. S11), a complex entity of DCC and carboxyl is first formed and subsequently attacked by hydroxyl function groups. Owing to the slow reaction rate, a small portion of the complex entities (less than 1%, estimated from the SIMS data below) still presents on the silicon surface, resulting in the incorporation of nitrogen atoms to the doping process. The presence of nitrogen has a profound implication for the electrical property of the doped samples^23^.

Atomic force microscope (AFM) was employed to visualize the morphology of PG modified silicon surface. The AFM images (SI Fig. S12) clearly show that the surface roughness has been significantly increased after immobilization of PG molecules, compared to the un-modified silicon surface (SI Fig. S13). This observation is consistent with XPS (Fig. 5) and infrared radiation (IR) transmission spectrum (see SI Figs S14–S15 and the related discussions).

### Molecular Doping in Silicon

After the polyglycerol grafting, the spin-on-glass (SOG) gel was spin-coated on the silicon surfaces to prevent the possible contamination from ambient environment. The samples were annealed by rapid thermal process (RTP) with a fast ramp of 200 °C/sec from room temperature to 1050 °C in inert atmosphere (N_2_). To drive the dopants into silicon, the samples were kept at 1050 °C for 30 s. After annealing, the doped silicon wafers were cleaned in the buffered oxide etchant (BOE) solution (6:1) to remove the silicon dioxide capping layer. Van der Pauw method was applied to measure the sheet resistance of the samples after metal contacts deposited by thermal evaporation^24^. Table 2 shows the measured sheet resistances R_m_ (second row, obtained using Equation II in SI). The current *vs* voltage (*I-V*) curves for the measured resistances are shown in the SI Figs S16–S19. Note that the intrinsic silicon wafer is 500 μm thick and the doping or possible contamination is located in the very top thin layer (<100 nm) underneath the surface. When electric field is applied in the lateral direction, current flows through the substrate bulk and the thin surface layer. Therefore, the measured sheet resistance (R_m_) is the combined resistance of the bulk (R_b_) and the top surface resistor (R_s_) which are electrically connected in parallel, i.e. R_m_ = R_b_ × R_s_/(R_b_ + R_s_). Given that the sheet resistance R_b_ for the as-purchased silicon substrate (cleaned using piranha and HF) is 411.0 kΩ, we calculate all the sheet resistance R_s_ for doping or contamination in the thin layer near the top surface from the measured sheet resistances R_m_ in the second row. The calculated results are shown in the third row of Table 2. The sheet resistance R_m_ for the blank sample that went through all the steps except monolayer modification is 199.8 kΩ, lower than 411.0 kΩ for the as-purchased sample. It means that certain contamination has brought in during the experimental processes. That part of contamination in the thin surface layer results in a sheet resistance (R_s_) of 388.9 kΩ. When the substrate is coated with the undecylenic acid (UA) monolayer, the sheet resistance (R_s_) still drops slightly to 341.2 kΩ although the monolayer does not contain any electrically active dopants. For the “2 days” sample that is reacting with the P carrier polyglycerol for 2 days, the sheet resistance (R_s_) contributed by the dopants is 100.1 kΩ which is further reduced to 34.5 kΩ when the reaction is extended to 11 days.

**Figure 6:**
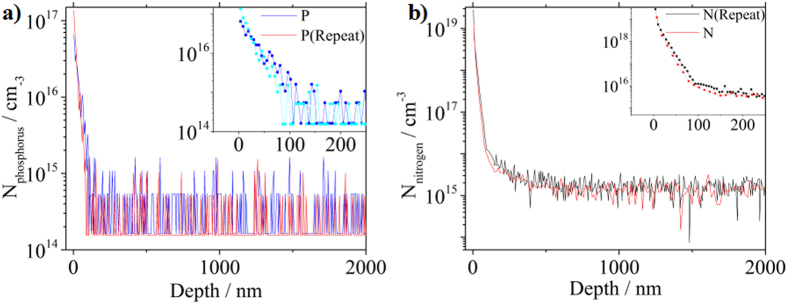
Dopant distribution profile of the “11 Days” sample from surface to bulk by SIMS: (**a**) Phosphorus and (**b**) Nitrogen. Inset: dopant concentration in logarithm scale.

Due to the fact that the distribution of dopants in the substrate is highly non-uniform, it is not reliable to find the dopant concentration from the sheet resistance unless the dopant distribution profile is acquired. Secondary ion mass spectrometry (SIMS) was performed to record the phosphorus distribution from the surface. Note that the profiles of other contaminants like carbon, oxygen and SiO moieties through monolayer doping method have been investigated by Yasuo Shimizu *et al*.^25^. Figure 6a shows the phosphorus profile for the “11 days” sample. The P concentration at the surface is approximately 10^17^ cm^−3^, 2 to 3 orders of magnitude lower than previous reports^10^,^26^, due to the large size of the polyglycerol molecules that limits the doping concentration. Most of the dopants are located in the top 100 nm thick layer near the surface. If we integrate all the P dopants and assume the activation rate 100% for the P dopants, the sheet resistance will be ~37.9 kΩ which is slightly higher than the experimental value 34.5 kΩ. It appears that the ionization rate of phosphorus dopants in our sample will be a little higher than 100%. After a critical investigation, we conclude that this is not due to the experimental data fluctuation, but probably caused by the nitrogen showed in the XPS spectrum in Fig. 2a and d. Previously, we found that nitrogen can also electrically dope silicon^23^. Nitrogen may play a role here in the electronic properties of the doped samples. SIMS was employed to characterize the distribution of nitrogen in silicon, as shown in Fig. 6b. The N concentration is surprisingly high (10^19^ cm^−3^ at the surface). The possible origin of nitrogen at such a high concentration is from the coupling reagent DCC as explained previously and surface absorbance of N_2_ gas.

**Figure 7:**
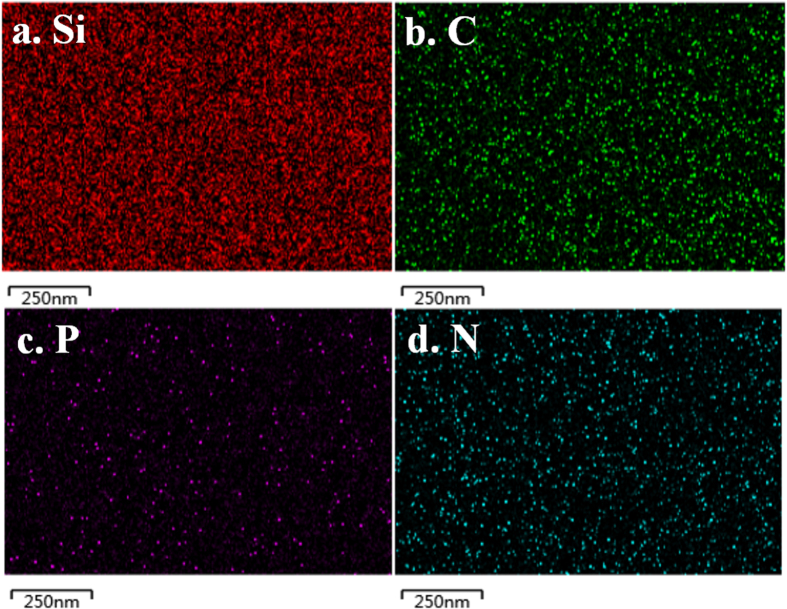
SEM/EDS element mapping in the planar surface of the macromolecule doped sample.

To analyze the distribution of dopant elements in the planar surface, energy dispersive X-ray spectroscopy (EDS, SI Fig. S20) and element mapping (Fig. 7 and SI Fig. S21) were employed to probe carbon, phosphorus and nitrogen elements. As expected, P, C and N elements have diffused into the silicon substrate. The distribution of these elements (P in particular) is discrete and uniform at micrometer scale, but follows a certain pattern at sub-micrometer scale (Fig. 7c), which is probably related to the morphology of the macromolecules on the silicon surface after the sample is dried.

**Figure 8:**
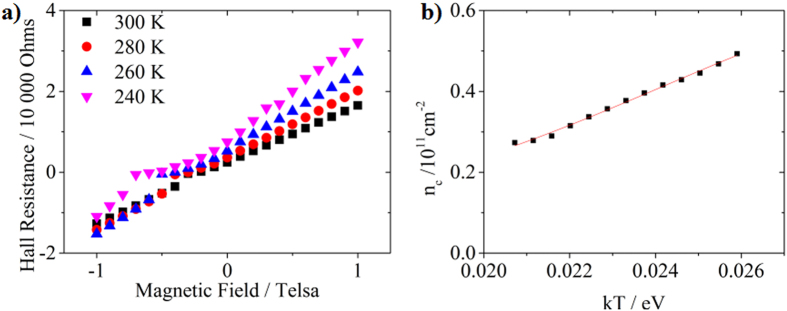
(**a**) Low-temperature Hall resistance of the “11 Days” sample. (**b**) Free electron concentration as a function of temperature.

The presence of nitrogen and phosphorus will both contribute to the electrical properties of the samples. To distinguish the electrical contribution of P and N dopants, low temperature Hall effect measurements are employed to characterize the “11 days” sample. The Hall resistance is mostly linear with the magnetic field at room temperature, but becomes increasingly nonlinear (at negative magnetic field in Fig. 8a) at low temperature probably because of the presence of magnetoresistance. Since the dopant distribution is highly non-uniform in the direction from the Si surface to the bulk, the electron concentration is calculated as per unit surface area (instead of per unit volume) from the Hall resistance (see Equation III in SI). The free electron concentration as a function of thermal energy *kT (k* as Boltzmann constant and *T* as absolute temperature) is presented in Fig. 8b. Data lower than 0.021 eV (*T* ≈ 230 K) are not included because the sample resistance is increased over the limit of the physical property measurement system (PPMS). It is known that the ionization energy of nitrogen (~189 meV) in silicon is much higher than that of phosphorus (~45 meV)^27^. The electrically active phosphorus dopants remain complete ionization when the temperature is higher than 230 K. The drop of the free electron concentration is solely due to the decrease of the nitrogen ionization rate (Fig. 8b). Based on our previous derivation (also see Equation IV in SI), the electron concentration per unit area *n*_c_ as a function of thermal energy *kT* follows the equation below:


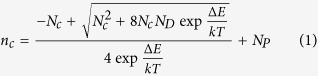


where *N*_c_ is the effective density of states function which is defined as 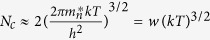 with *w* being the constant related to the band structure of the semiconductor, *N*_D_ the concentration of nitrogen donors, N_P_ the concentration of phosphorus donors, and Δ*E* the activation energy which is equal to (*E*_c_ − *E*_d_) with *E*_c_ and *E*_d_ being the conductance band edge and the donor energy level, respectively.

By fitting the above equation to the data in Fig. 8b, we obtain the activation energy of the nitrogen dopants, the concentration of electrically active nitrogen and phosphorus as listed in Table 3. The activation energy of nitrogen is 210 meV, a little higher than 189 meV that we previously reported^23^. The concentration of electrically active nitrogen is 6.79 × 10^10^ cm^−2^, approximately 6 times higher than that of phosphorus (1.14 × 10^10^ cm^−2^). From the SIMS data (insets of Fig. 6a and b), the average nitrogen and phosphorus concentration within 100 nm below the surface is 1.40 × 10^13^ cm^−2^ and 1.64 × 10^11^ cm^−2^, respectively. Accordingly, the activation rate is 0.49% for nitrogen and 7% for phosphorus. The relatively low ionization of phosphorus is likely due to the fact that nitrogen can electrically retard phosphorus by forming N-P complex entities.

To improve the ionization rate of phosphorus, we modify the experimental processes that possibly introduce nitrogen. First, the macromolecules are directly immobilized on the silicon surfaces without the coupling agent DCC. Second, the samples are annealed in Ar instead of N_2_. SIMS and low-temperature Hall measurements are performed again to characterize the ionization rate. Unexpectedly, a large quantity of nitrogen is still observed in the sample and the ionization rate of phosphorus continues to remain low. In this case, the source of nitrogen is mostly likely from the physical absorption of N_2_ from the air since nitrogen is also detected near the surface of as-purchased intrinsic silicon substrate (see SI Fig. S22 and the related discussions). It is known that the triple bond in N_2_ molecules is extremely strong and a temperature 1050 °C is not possible to break the bond^28^. It is unlikely that N_2_ could form N-P complexities to retard the ionization of phosphorus. Recently, we performed deep level transient spectroscopic (DLTS) measurements^29^ on similar samples, and found that carbon can form deep levels in silicon. The existence of deep levels will trap the free electrons provided by the phosphorus dopants, creating a retardation observation of dopants. Fortunately, only a small portion of carbon atoms is interstitial, which creates deep energy levels. Therefore, for highly doped samples, the ionization rate only drops slightly^7^,^8^,^9^. For lightly doped samples, however, the ionization rate will significantly reduce like the case investigated in this Letter because the P to C stoichiometric ratio is low. This observation is consistent with the previous results in which the resistivity of lightly doped samples is unexpectedly high^7^. To reduce the carbon defects, we developed a new process by annealing the sample in O_2_ at 550 °C for 5 hours, right before the rapid thermal annealing at 1050 °C. The polyglycerol macromolecules are burned into gaseous CO_2_ and CO that will escape out of the SOG coating, minimizing the co-doping of carbon with phosphorus. Low-temperature Hall measurements were conducted on the sample, revealing a significantly improved ionization rate of phosphorus (see SI Fig. S23).

## Conclusions

In conclusion, we have successfully synthesized and characterized super-branched polyglycerol macromolecules that are potentially ideal carrier candidates for single dopants control. The macromolecules each carrying one phosphorus atom in the core are immobilized on the silicon surface by forming covalent bonds with the pre-modified silicon surfaces. Electrical and SIMS measurements at room temperature showed that the apparent ionization rate of phosphorus dopants is approximately 100%. A careful investigation by low temperature Hall measurements revealed that nitrogen dopants also partially contribute to the conductivity of the doped samples, and that the ionization of phosphorus is actually only 7%. New experiments were devised to remove the nitrogen and investigate the dopant ionization. The results indicate that the ionization process is more complicated than expected, in which interstitial carbons may also play an important role.

## Experimental Section

### Materials

FZ intrinsic Silicon <100> wafers were purchased from Institute of Electronic Materials Technology (ITME). The wafer thickness is 500 ± 20 μm and the resistivity is higher than 10 kΩ·cm. The tris(4-methoxyphenyl)phosphine and undecylenic acid (>97.5%) were bought from TCI Shanghai Co., Ltd and J&K company, respectively. Other reagents were analytic grade without special treating. Before annealing process, all samples were cleaned by CMOS grade solvents.

### Synthesis of Tris(4-hydroxylphenyl)phosphine Oxide

Tris(4-methoxyphenyl)phosphine (503 mg, 1.43 mmol) was dissolved in the 15 mL of EtOAc and then hydrogen peroxide solution (30%(w/w), 0.3 mL, eq. 5) was added slowly to the solution dropwise. The mixture was stirred for 2 hours at room temperature. After the reaction finished, EtOAc was removed under vacuum pressure, after which the residue was extracted by EtOAc, collected and dried by anhydrous Na_2_SO_4_. In the end, the organic solvent was removed under vacuum pressure. The crude product was applied for the next step without purification.

Tris(4-methoxyphenyl)phosphine oxide was dissolved in 30 mL dichloromethane, and then BBr_3_ solution (1 M, 15 mL) was added dropwise slowly to the solution under an ice cooling bath. Afterwards the mixture was stirred at room temperature for 5 hours. The reaction was quenched by adding methanol (15 mL). The purification process is similar to the previous oxidation steps, and the crude product was purified by flash chromatography to get tris(4-hydroxyphenly)phosphine oxide(343 mg, 1.05 mmol, 73.6%). ^1^H NMR (600 MHz, DMSO-*d*_*6*_): δ 10.11(s, 1H), 7.35(*dd, J* = *12 12* *Hz*, 2H), 6.87(*dd, J* = *6 6* *Hz*, 2 H); MS(ESI) found m/z: 325.0635, 326.0701.

### Synthesis of Polyglycerol

Tris(4-hydroxyphenyl)phosphine oxide (97 mg, 0.30 mmol) was dissolved in the 10 mL 1, 4-dioxane. Then NaH (250 mg, 60% in mineral oil) was added to the solution and stirred for 15 min at 90 °C. Glycerol (10 mL) was added dropwise slowly to the solution (more than 3 s between the two drops) and stirred for 12 h. After the reaction, the gel was washed by 1, 4-dioxane (10 mL * 3). The residue gel was dissolved in 20 mL de-ionized water and purified by the dialysis bag(molecular weight cutoff > 1000 g·mol^−1^). The Gel Permeation Chromatography results showed that *M*_w_ = 10296.4 g·mol^−1^, *M*_n_ = 5452.2 g·mol^−1^, *M*_z_ = 22204 g·mol^−1^, *M*_w_/*M*_n_ = 1.8885. ^31^P NMR (400 MHz): δ 26.33 (TPP as inner standard).

### Preparation of Undecylenic Acid Monolayer

The silicon wafer was cut into pieces of 15 mm × 15 mm. The silicon pieces were sonicated in acetone, ethanol and water, respectively, each for 10 min. The pieces were oxidized in the Piranha solution (sulfuric acid: 30% hydrogen peroxide = 3:1, 85 °C) for 45 min, and then washed by de-ionized water. The oxidized silicon pieces were etched in the 2.5% HF solution for 90 s, washed by de-ionized water for several times, and then blown dry by nitrogen gas.

All the reaction tubes were dried at 140 °C for 3 hours. Undecylenic acid was deoxygenated by bubbling argon gas for 30 min. Before the monolayer preparation, quartz tubes were de-oxygenated and de-water under argon atmosphere for 30 min. The undecylenic acid and the fresh hydrogen-terminated silicon pieces were added to the quartz tube at room temperature under UV light (3.6 w·cm^−2^) overnight. After the reaction, the silicon pieces were washed by ethyl acetate and CMOS ethanol and then blown dry by nitrogen gas. The undecylenic acid monolayer was characterized by XPS.

### Polyglycerol Binding to the Silicon Surface

Polyglycerol (100 mg) was dissolved in 10 mL DMF. Then the silicon pieces were immersed in the solution followed by adding DCC (1 g) and DMAP (100 mg) into the solution. After the reaction finished, the silicon pieces were washed by EtOAc, EtOH, and DI water and blown dry by nitrogen gas.

### Silicon Dioxide Capping Layer and Thermal Annealing

SiO_2_ films were formed by spinning spin-on-glass (SOG, IC1-200) gel on the silicon pieces. Rapid thermal annealing of the samples was conducted at 1050 °C for 30 s with a temperature ramp of 200 °C/s. After annealing, the Si samples were immersed again in BOE solution (6:1) to remove SiO_2_ film on the surfaces.

### Electrical Characterization

A home-made probe station equipped with four tungsten probes was used for van der Pauw measurements. Metal contacts on the four corners of the samples were deposited by evaporating 150-nm-thick Al films in the vacuum thermal evaporator (Angstrom Engineering, Canada). Two Keithley 2400 sourcemeters were used to generate and collect current and voltage signals. Van der Pauw measurements were carried out by sweeping source voltage from −1 to 1 V while monitoring the currents and voltages. Low temperature Hall effect measurements were carried out in the physical property measurement system (PPMS) from Quantum Design Inc. (model PPMS-9T EC-II).

### IR and AFM measurement

Bruker ICON Atomic Force Microscope was applied to probe the sample surface in air. Infrared (IR) spectra of PG molecule were obtained using Fourier transform infrared spectromter Nicolet 6700 (Thermo Fisher). The IR transmission spectrum was obtained by Nicolet iN10 MX, Thermo Fisher company, and the scanning times was set at 256, while other parameters were set as default.

### TEM and SEM measurement

Zeiss Ultra Plus Field Emission Scanning Electron Microscope was employed to take the TEM images of a sample slide that was cut by focus ion beam (FIB). For EDS mapping measurement, the acceleration voltage was 5 kV, and other parameter was set as default. SEM images were performed with JEOL 2000f.

## Additional Information

**How to cite this article:** Wu, H. *et al*. Controlled doping by self-assembled dendrimer-like macromolecules. *Sci. Rep.* **7**, 41299; doi: 10.1038/srep41299 (2017).

**Publisher's note:** Springer Nature remains neutral with regard to jurisdictional claims in published maps and institutional affiliations.

## Supplementary Material

Supplementary Information

## Figures and Tables

**Figure 1:**
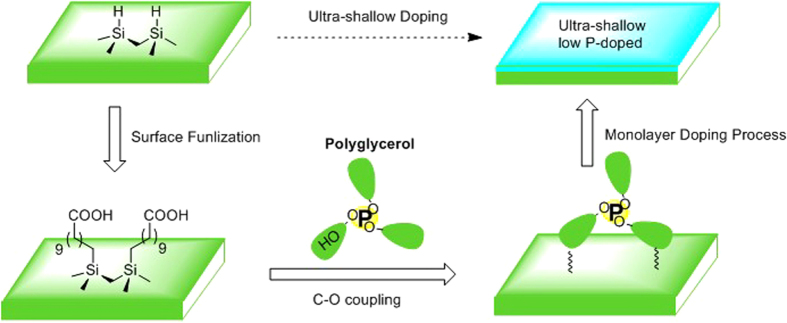
Dendrimer-like macromolecule doping protocol.

**Table 1:** The area of peaks in the IG ^13^C NMR spectrum.

Type	*L*_*13*_	*D*	*2L*_*14*_	*2D, 2T*	*L*_*13*_*, L*_*14*_	*T*	*L*_*13*_
Area	1	7.08	22.92	38.98	13.24	11.58	1.65

**Table 2:** Sheet resistance of silicon samples.

	Blank Sample	UA Sample	2 Days	11 Days
Measured Sheet Resistance (R_m_)	199.8 kΩ	126.0 kΩ	66.7 kΩ	29.4 kΩ
Calculated Sheet Resistance (R_s_) of Dopants/Contaminations	388.9 kΩ	341.2 kΩ	100.1 kΩ	34.5 kΩ

**Table 3:** Fitting results for the data in Fig. 8b.

Parameter	Value	Standard Error
N_d_ (10^11^ cm^−2^)	0.679	0.046
N_P_ (10^11^ cm^−2^)	0.114	0.025
ΔE (eV)	0.210	0.008
