# Supplementary material for: Controlled doping by self-assembled dendrimer-like macromolecules
Source: Sci Rep. 2017 Feb 1;7:41299. doi: 10.1038/srep41299 (PMC5286916; doi:10.1038/srep41299)
Supplement: Supplementary Information [file srep41299-s1.pdf]

# Controlled doping by self-assembled dendrimer-like macromolecules

# (Supporting Information)

Haigang Wu[a,b], Bin Guan[b] , Yingri Sun[b], Yiping Zhu[c] and Yaping Dan[b*]

[a]School of Biomedical Engineering, Shanghai Jiao Tong University, Shanghai, China 200240

[b]University of Michigan- Shanghai Jiao Tong University Joint Institute, Shanghai Jiao Tong University, Shanghai, China 200240

[c]Key Laboratory of Polar Materials and Devices, Ministry of Education, and Department of Electronic Engineering, East China Normal University, 500 Dongchuan Road, Shanghai 200241, China

*To whom correspondence should be addressed: yaping.dan@sjtu.edu.cn

The supporting information contains 23 figures and 4 equations.

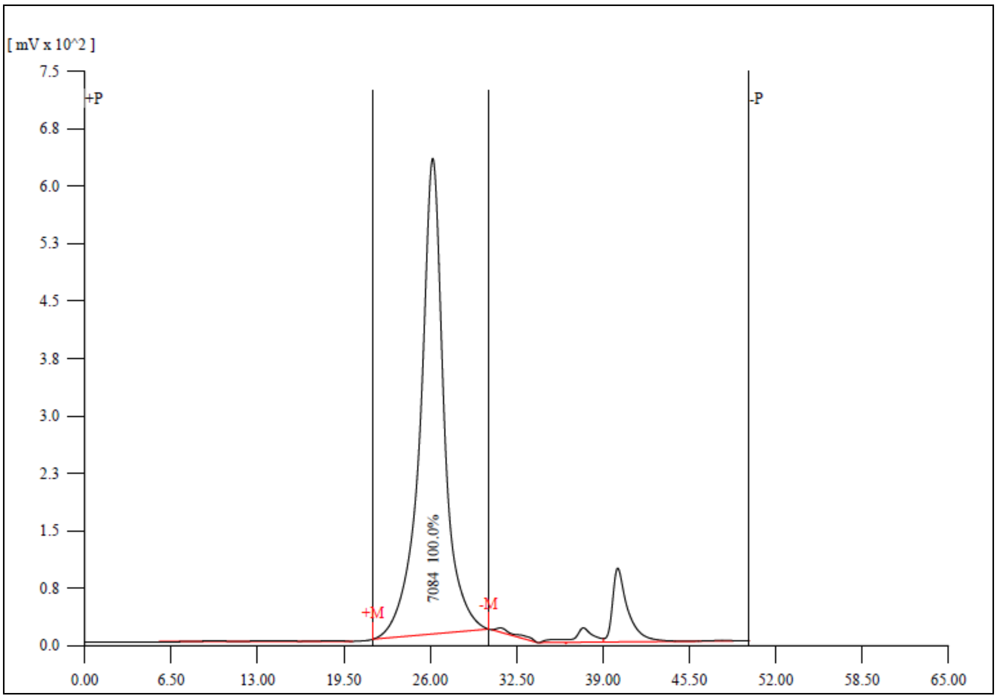

Figure S1. GPC results of polyglycerol (X-axis is time and Y-axis is signal strength voltage). Water is the mobile phase and the peaks at around 39.00 min are related to some impurity substances from water.

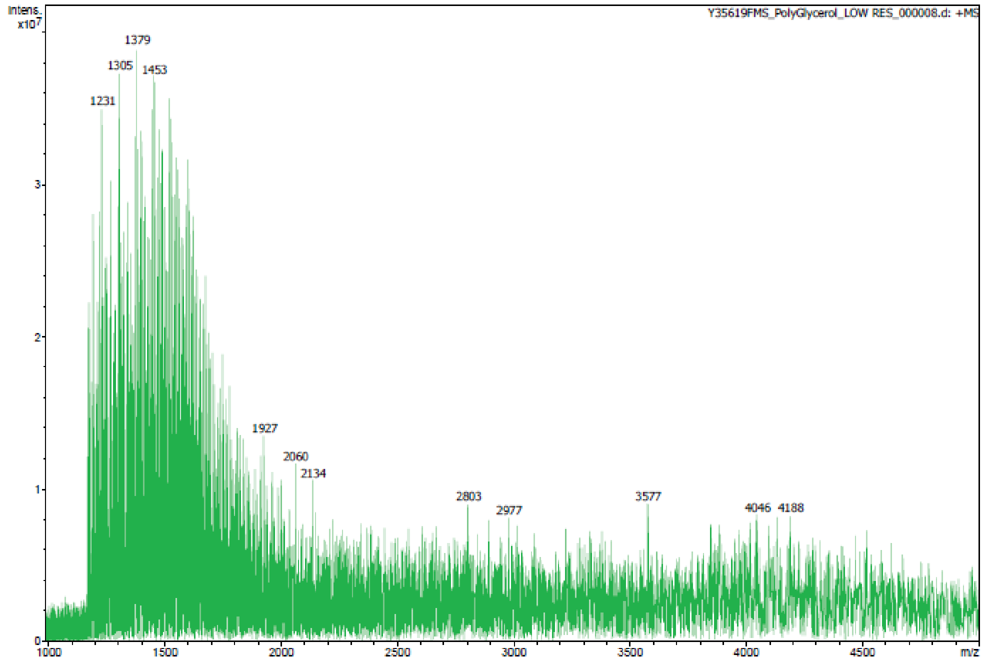


Figure S2. ESI-MS spectra of Polyglycerol at the molecular weight of 1000 to 5000. Owing to the limitation of ESI equipment, the polyglycerol molecules with large molecular weight may not be ionized.

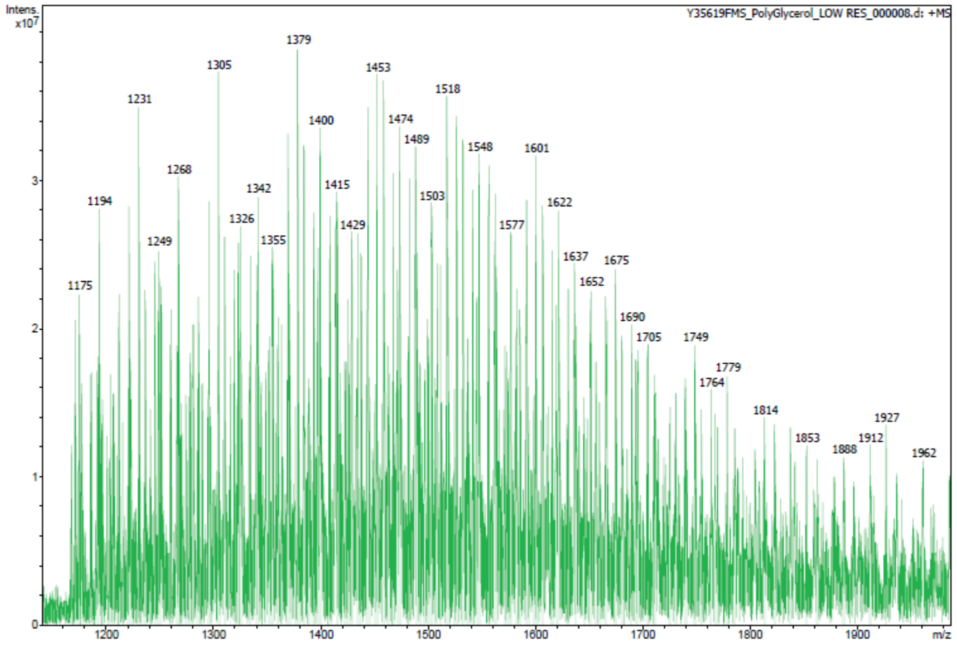


Figure S3. MS-ESI spectra of Polyglycerol.

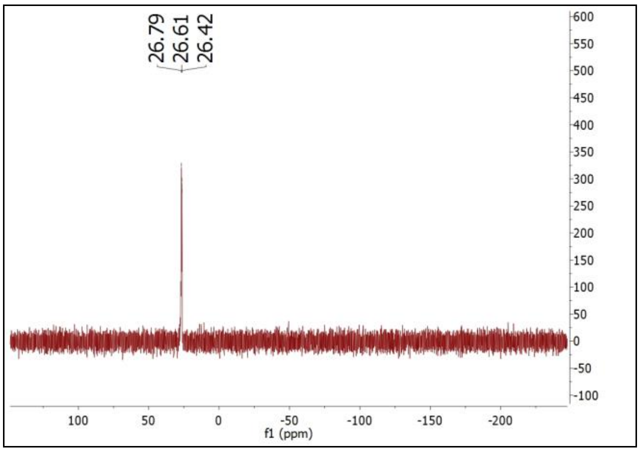


Figure S4. $^{31}P$ NMR spectra of polyglycerol (TPP as inner standard)

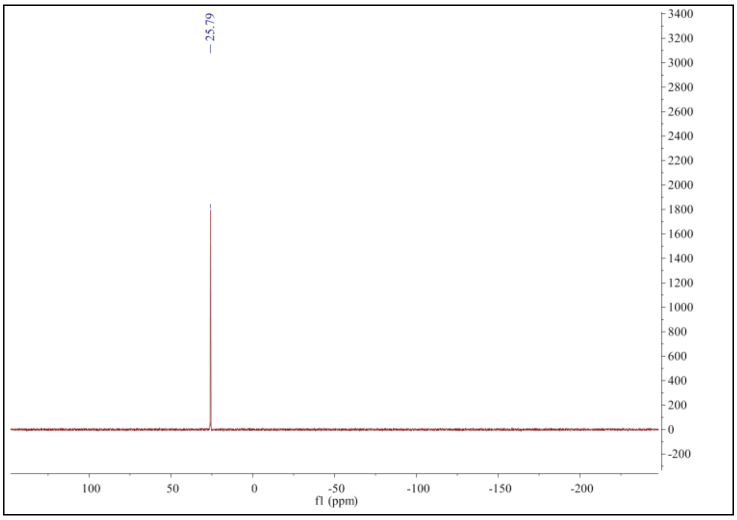


Figure S5. $^{31}P$ NMR of tris(4-hydroxyphenyl)phosphine oxide. Triphenylphosphine (TPP) is as the inner standard.

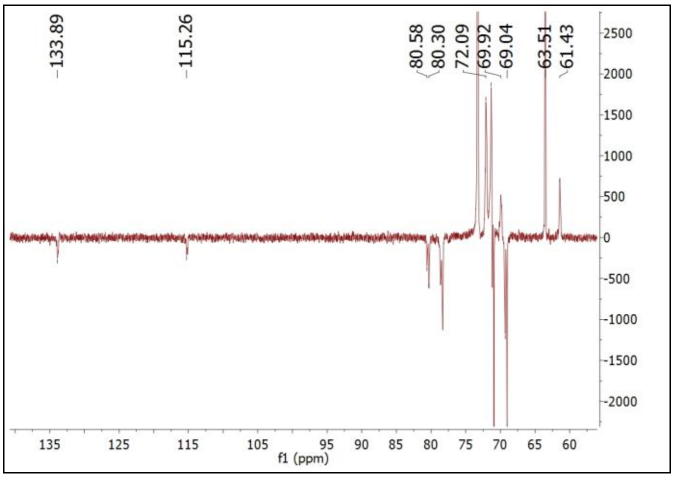


Figure S6. $^{13}C$ DEPT 135 °NMR of polyglycerol. The negative signals of $^{13}C$ NMR chemical shift at 115.26 ppm and 133.89 ppm are from phenyl carbons of the tris(4-hydroxylphenyl)-phosphine oxide in the polyglycerol.

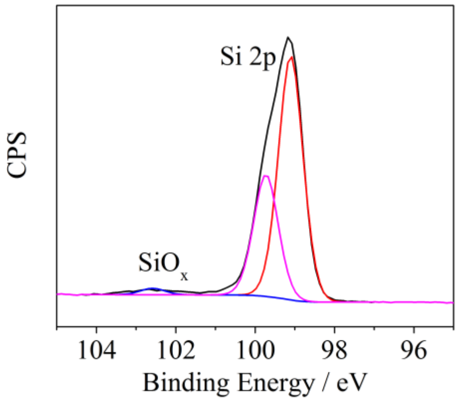


Figure S7. XPS survey spectrum of sample "11 days" at Si 2p region.

The $SiO_x$ coverage can be calculated on basis of the theory in *Journal of Physical Chemistry B* 106 (2002): 3639-3656. The theory first calculates the integral ratio of Si-$O_x$ to Si 2p peak in XPS spectra if 100% of the surface Si atoms are oxidized, which is given by

$$\frac{I_{Si(-Ox)}}{I_{Si,2p}} = \frac{n_{Si,surf}}{n_{Si,bulk}\, l_{Si} - n_{Si,surf}}$$

where $n_{si,surf}$ is the surface density of Si atoms ($7.8\times10^{14}$ $cm^{-2}$ for Si(111) and $6.9\times10^{14}$ $cm^{-2}$ for Si(100)), $n_{Si,bulk}$ is the atomic number density of Si atoms ($5.0\times10^{22}$ $cm^{-3}$), and $l_{Si}$ is the escape depth equal to $\lambda_{Si}\times\sin(90°)$ in which $\lambda_{Si}$ is 1.6 nm and 90 °is the angle between the detector and sample surface for our instrument.

The calculated result indicates that the integral area ratio of Si(-$O_x$) to Si 2p peak shall be 0.094 if a monolayer of $SiO_x$ is formed on the Si surfaces (all the surface Si atoms are oxidized). Our measured ratio (Fig.S7 above) is only 0.018, meaning that approximately 0.2 monolayer of $SiO_x$ is formed on the surface of our undecylenic acid modified sample.

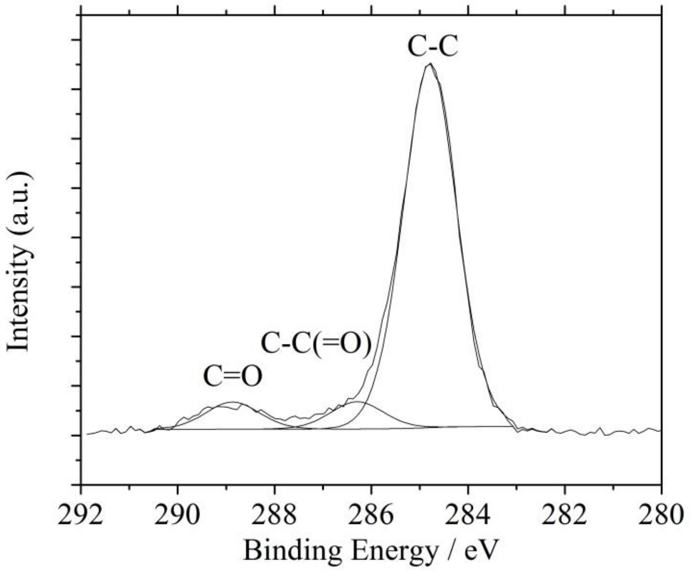


Figure S8. XPS spectrum of C 1s taken for undecylenic acid monolayer on silicon surface. Three peaks are fitting in the curve: C=O, C-C(=O) and C-C.

The characteristic C-C(O) peak is 0.98 times of the C=O peak in terms of integral area, which is close to the stoichiometric ratio (1:1) of C-C(O) to C=O in undecylenic acid molecules.

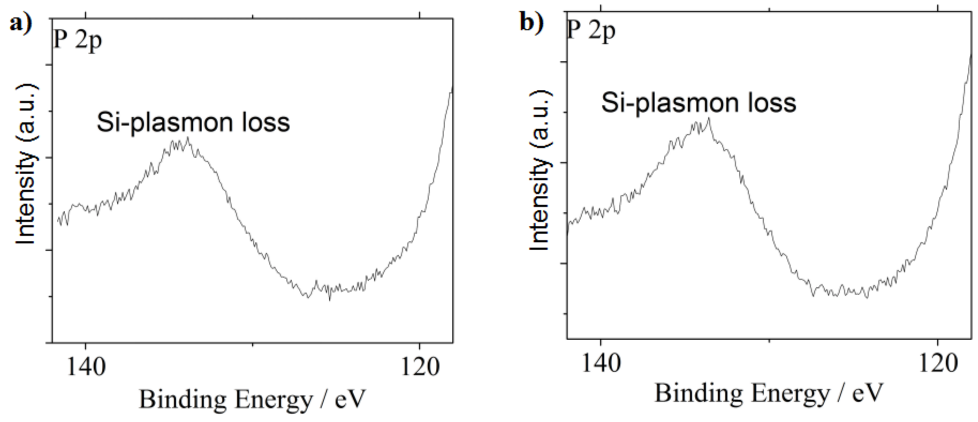


Figure S9. XPS survey spectra of samples at P 2p region for (a) the control sample (with DCC) and (b) the sample "3" with DCC for 11 days reaction time.

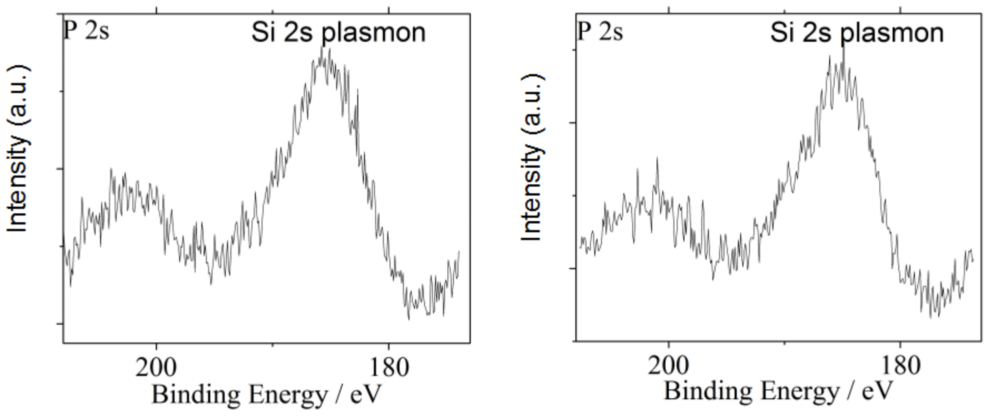


Figure S10. XPS survey spectra of samples at P 2s region for (left) the control sample (with DCC) and (right) the sample "3" with DCC for 11 days reaction time.

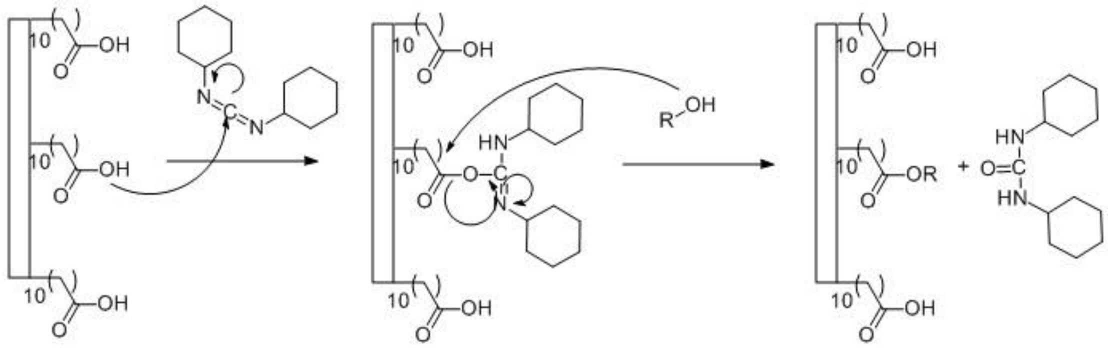


Figure S11. Mechanism of ester formation process with DCC coupling reagent.

During the coupling process, only two coupling reagents are used, i.e. DCC (Dicyclohexyl-carbodiimide) and DMAP (4-dimethylaminopyridine). The binding energy of N peak in the XPS spectra is 400.3 eV, which is different from N in DCC (399.00 eV) and N in DMAP (399.50 eV of dimethylamino and 399.00 eV of pyridine)[1]. As a result, the nitrogen doped in silicon must come from the intermediate of DCC and carboxyl group.

[1] NIST X-ray Photoelectron Spectroscopy Database, Version 4.1 (National Institute of Standards and Technology, Gaithersburg, 2012); http://srdata.nist.gov/xps/.

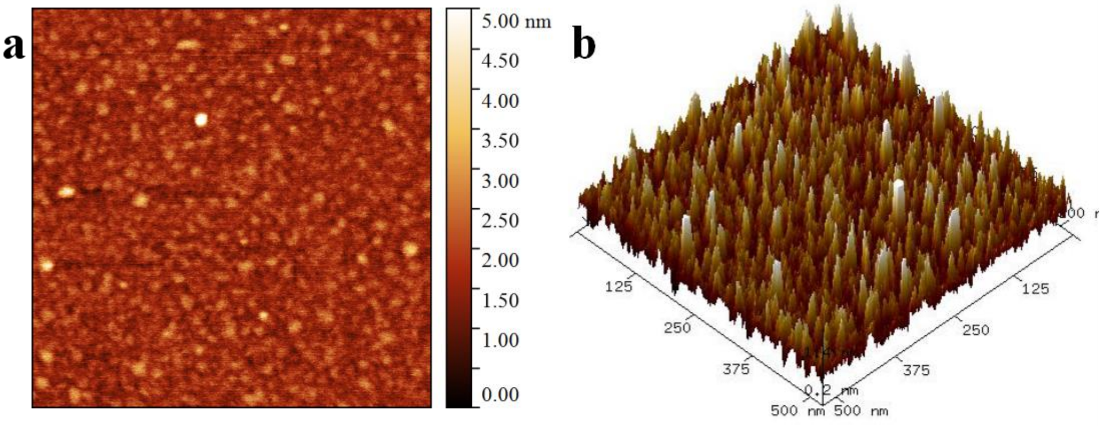


Figure S12. AFM images of silicon surface modified with polyglycerol macromolecules.

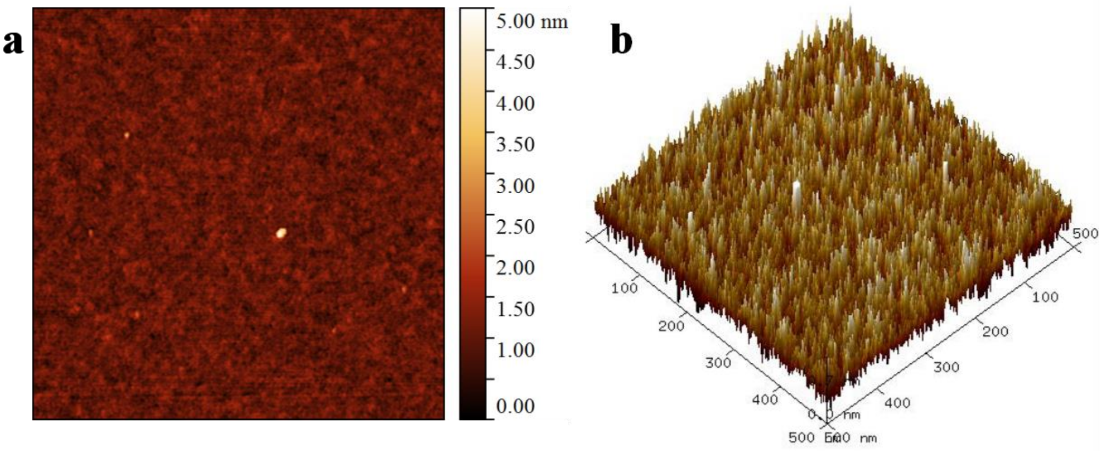


Figure S13. AFM images of un-modified silicon surface.

Compared with Fig.S12, the roughness of macromolecule-modified sample significantly increases, which is caused by the PG molecules.

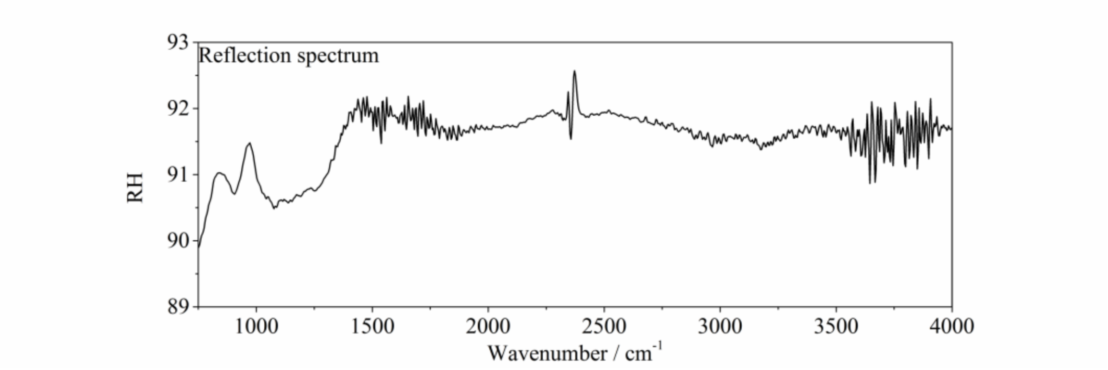


Figure S14. IR reflection spectra of macromolecule-modified sample. The CH modes are too weak to identify.

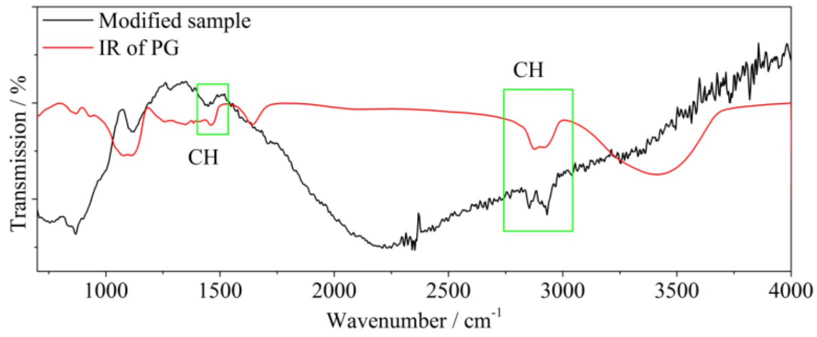


.Figure S15. IR transmission spectra of PG macromolecules (red) and PG-modified silicon (black).

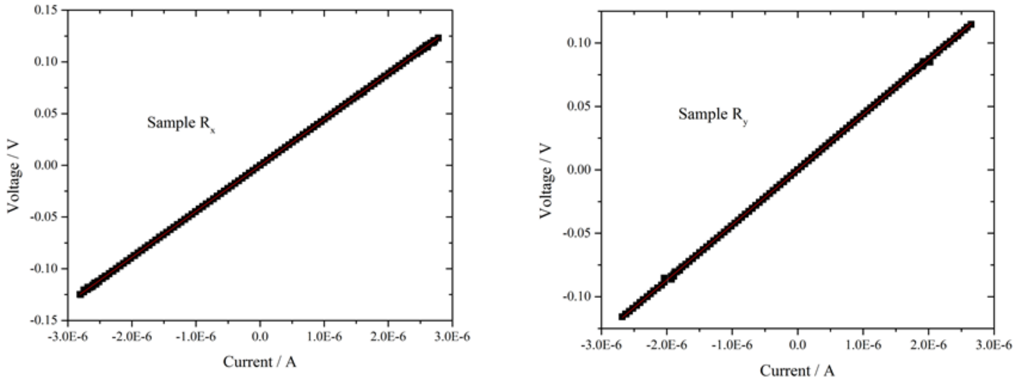


Figure S16. *I*-*V* curves of the blank sample

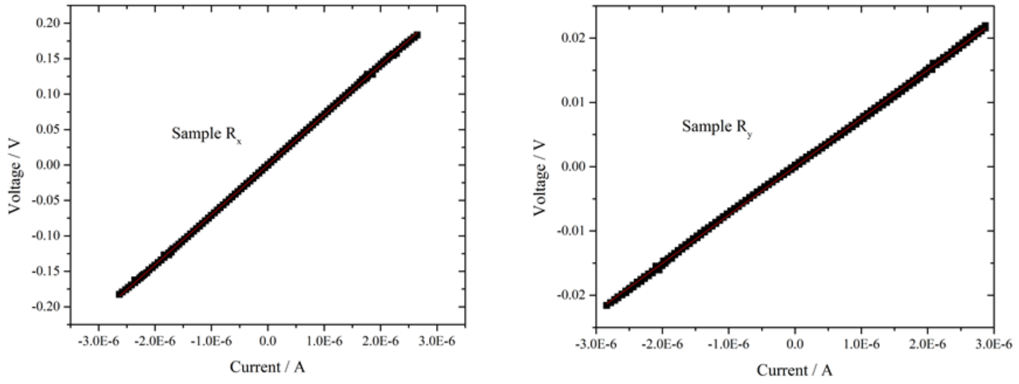


Figure S17. *I*-*V* curves of undecylenic acid monolayer sample

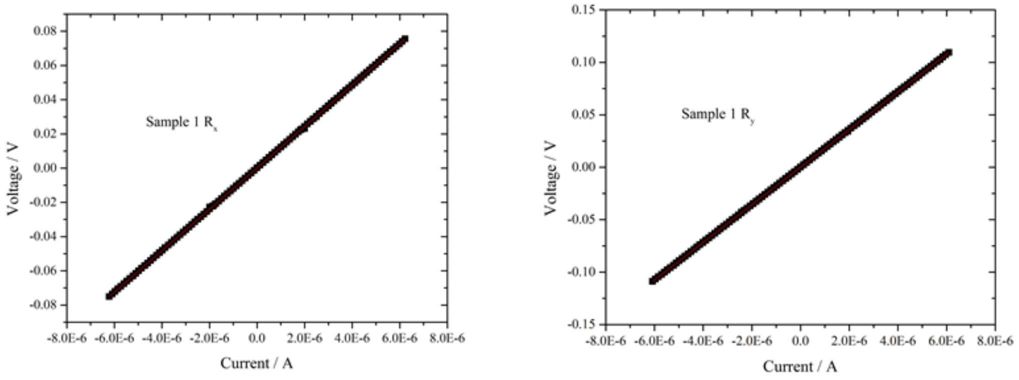


Figure S18. *I*-*V* curves of the “2 days” sample

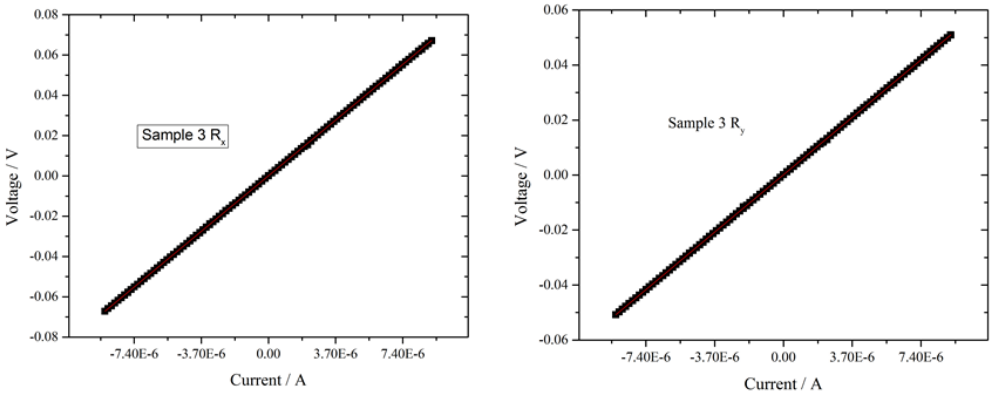


Figure S19. *I-V* curves of the "11 days" sample

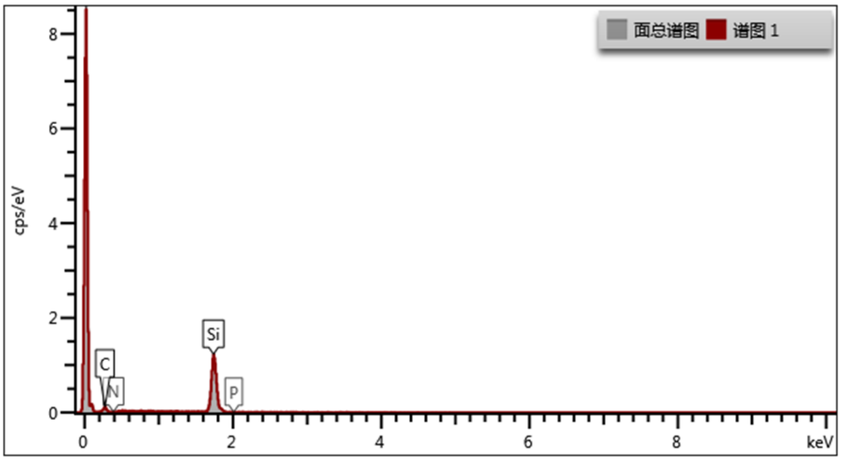


Figure S20. EDS elemental spectra of the doped silicon sample.

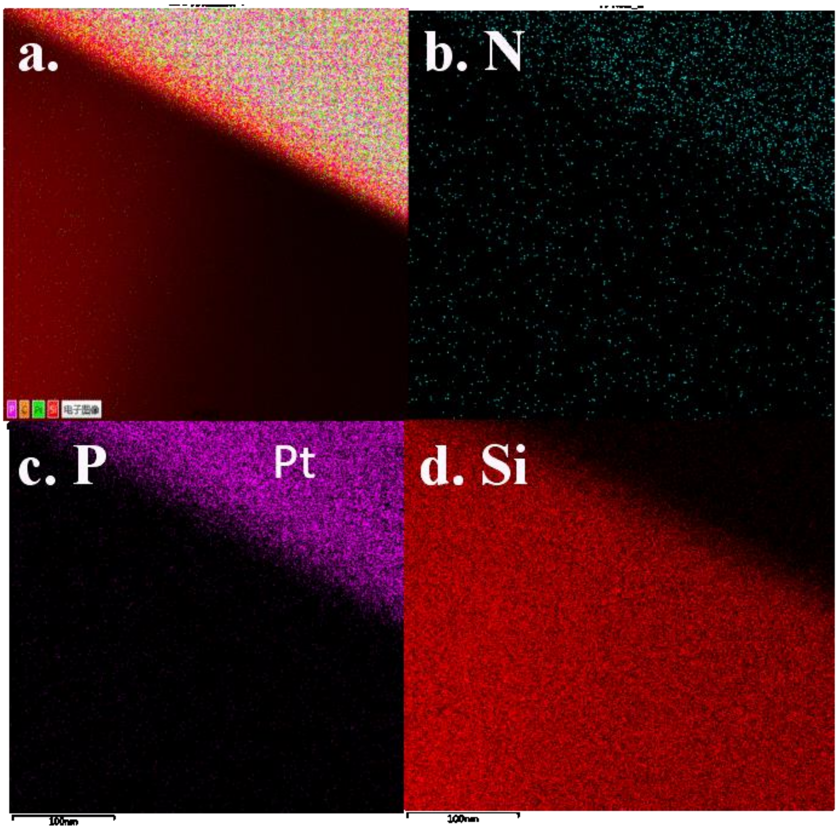


Figure S21. Cross-sectional element mapping of the monolayer doped sample by HAADF-STEM. To do TEM, a thin slab of the sample is cut by focused ion beam (FIB), during which a layer of Pt is deposited on the sample surface. However, P element mapping is problematic because the k α 1 signal for P and Pt are located at the same energy position, resulting in the same purple dots in Panel c.

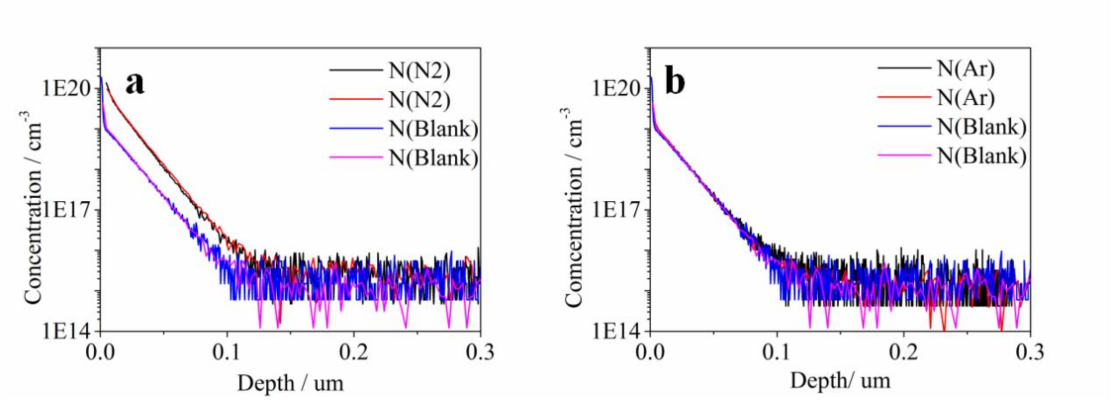


Figure S22. SIMS N distribution from surface to bulk for samples annealed in N2 and Ar enviroment. Surprisingly, N elements are shown up in the blank sample which is undoped silicon as purchased. This is due to the physical absorption of N2 on the sample surface.

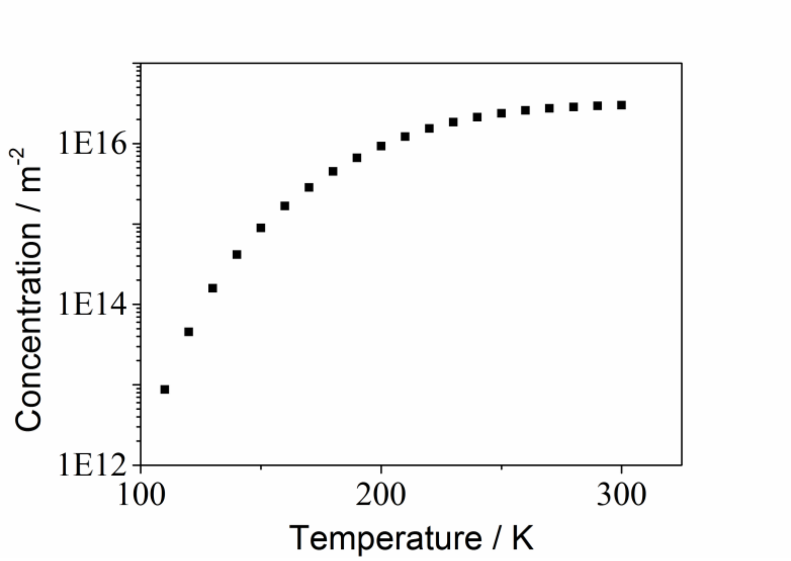


Figure S23. Hall effect of samples pre-treated with oxygen ambient (550 ℃ for 5h).

In order to remove the carbon contaminant, the PG modified silicon sample was pre-treated at 550 ℃ in oxygen ambient for 5h. After the pre-treatment, the silicon sample was annealed at 1050 ℃ in vacuum for 30s. This sample was measured by the PPMS to find the concentration of electrically active dopants. The results indicate that oxygen pretreatment significantly improves the phosphorus activation rate.

**Equation I:**

Equation I:

$$M_w = \frac{\sum m_i M_i}{\sum m_i} = \frac{\sum niM_i^2}{\sum n_i M_i} = \sum w^m{}_i M_i$$

$$M_n = \frac{\sum n_i M_i}{\sum n_i} = \sum w^n{}_i M_i$$

$\boldsymbol{M_i}$, Molecular Weight; $\boldsymbol{m_i}$, weight of molecules; $\boldsymbol{n_i}$, molar of molecules;$\boldsymbol{W^m{}_i}$, weight percentage ratio of molecules; $\boldsymbol{W^n{}_i}$, molar percentage ratio of molecules.

**Equation II**

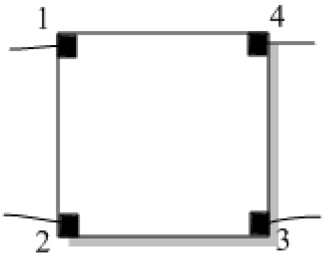


$$\exp\left(-\frac{\pi R_x}{R_s}\right) + \exp\left(-\frac{\pi R_y}{R_s}\right) = 1$$

$$R_x = \frac{1}{2}(R_{12,34} + R_{34,12}),\ R_y = \frac{1}{2}(R_{14,23} + R_{23,14})$$

$$Rs = \frac{\pi d}{\ln 2} \times \frac{R_x + R_y}{2} \times f$$

The parameter f chooses a value from the following curve.

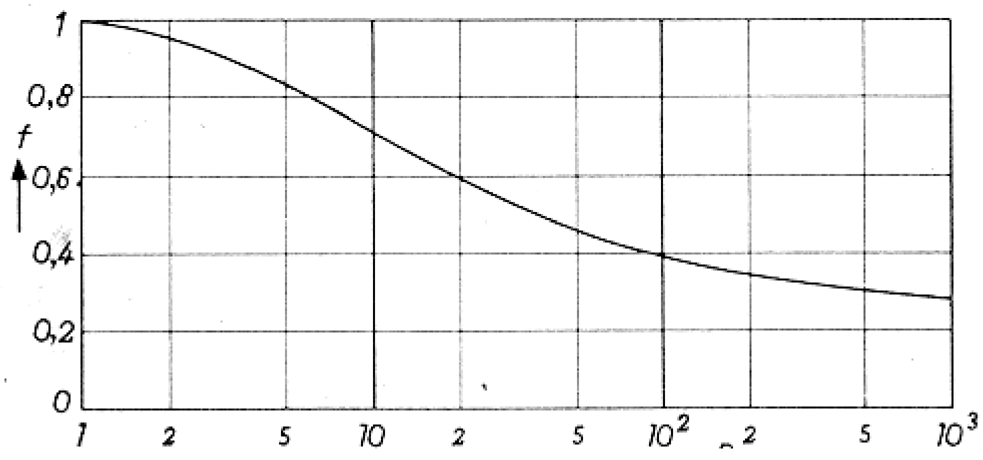


**Ration of $R_x$ to $R_y$**

**Reference: L. J. van der Pauw, A method of measuring specific resistivity and Hall effect of discs of arbitrary shape, Philips Res. Repts. 13, 1-9, 1958 (No.1).**

**Equation III**

Low-temperature Hall Effect Measurement

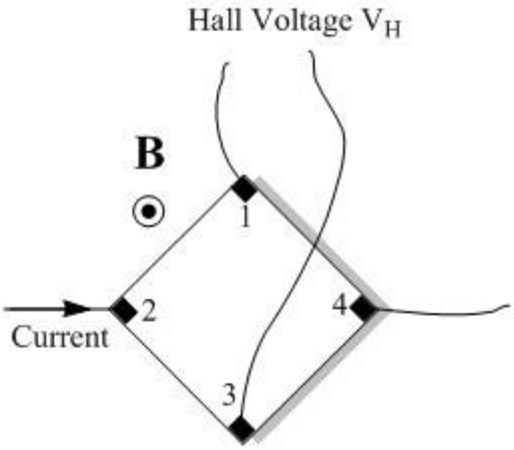


Hall Effect measurement protocol. DC current flowed from point 2 to point 4 and Hall voltage was measured between point 1 and point 3. The magnetic field was perpendicular to the paper surface.

$$V_H = \frac{R_H IB}{d}$$

$$\frac{V_H}{I} = \frac{R_H}{d} B = -\frac{1}{ned} B = -\frac{1}{\int_0^d n(x) edx} B = -\frac{1}{e\int_0^d n(x) dx} B = -\frac{B}{en_c}$$ Hall Effect equation.

**Equation IV**

**The equation about dopants energy level in nitrogen doped silicon**

For the N-doped silicon bulk, according to the charge neutrality, we have:

$$n_0 = N_d^+ + p_0 \quad (1)$$

Where $n_o$ is the electron concentration, $N^+_d$ is the average concentration of ionized dopants and $p_0$ is the hole concentration.

And in the n-type semiconductor, it follows:

$$n_0 \gg p_0 \quad (2)$$

Thus, we have

$$n_0 \approx N_d^+ \quad (3)$$

Also, it is known that the electron concentration is dependent upon the temperature, according with the equation

$$n_0 = N_c \exp\left(\frac{E_F - E_c}{kT}\right) \quad (4)$$

Where $N_c$ is the effective density of states function, $N_c \approx w*(kT)^{3/2}$ in which W is constant for the silicon semiconductor, $E_F$ is the Fermi level, $E_c$ is the conductance band edge.

And the average concentration of ionized dopants $N^+_d$ follows the equation

$$N_d^+ = \frac{N_d}{1 + 2\exp\left(\frac{E_F - E_d}{kT}\right)} \quad (5)$$

Where $E_d$ is the donor energy level.

Combined eq.(1)~eq.(5), and by solving the 2-order equation (eq.7) related to $n_0$, we have the equation (8)

$$N_c \exp\left(\frac{E_F - E_c}{kT}\right) = \frac{N_d}{1 + 2\exp\left(\frac{E_F - E_d}{kT}\right)} = \frac{N_d}{1 + 2\exp\left(\frac{E_F - E_c + E_c - E_d}{kT}\right)} \quad (6)$$

$$\exp\left(\frac{E_F - E_c}{kT}\right) = \frac{n_0}{N_c} = \frac{N_d}{1 + 2\frac{n_0}{N_c}\exp\left(\frac{E_a}{kT}\right)} \quad (7)$$

Where $E_a$ is the activation energy, with $E_a = E_c - E_d$.

$$n_0 = \frac{-N_c + \sqrt{N_c{}^2 - 8N_cN_d\exp\left(\frac{E_a}{kT}\right)}}{4\exp\left(\frac{E_a}{kT}\right)} \quad (8)$$

Note that phosphorus atoms remain complete ionization and the average concentration of completely ionized P donors, $N_p$, remains constant in the temperature range of the measurement. Hence, the electron concentration of the the N and P doped sample is shown as follows.

$$n_0 = \frac{-N_c + \sqrt{{N_c}^2 - 8N_cN_d\exp\left(\frac{E_a}{kT}\right)}}{4\exp\left(\frac{E_a}{kT}\right)} + N_P \quad (9)$$
